# The Work-Related Basic Need Satisfaction Scale: An Italian Validation

**DOI:** 10.3389/fpsyg.2018.01859

**Published:** 2018-10-02

**Authors:** Daiana Colledani, Dora Capozza, Rossella Falvo, Gian Antonio Di Bernardo

**Affiliations:** ^1^Department of Philosophy, Sociology, Education and Applied Psychology, School of Psychology, University of Padua, Padua, Italy; ^2^Department of Education and Humanities, University of Modena and Reggio Emilia, Modena, Italy

**Keywords:** W-BNS scale, basic needs, Self-Determination Theory, organizational commitment, KUT measure

## Abstract

The main goal of the present study was to validate the Work-related Basic Need Satisfaction (W-BNS) scale in the Italian social context. Three studies were carried out. Study 1 was conducted on two samples of employees. Exploratory factor analysis and parallel analysis were run on the first sample, whereas confirmatory factor analyses were run on the second. Results supported the three-dimensional structure of the W-BNS scale. Study 2 was conducted on a third sample of employees. Results supported the construct validity of the scale, by showing that needs for competence, autonomy, and relatedness were associated with job resources (social support, job autonomy, professional growth), low burnout, and job attitudes (job satisfaction, turnover intentions, commitment). In addition, results showed that responses to the scale were not affected by social desirability bias. Study 3 was conducted to evaluate the nomological validity of the scale (the sample grouped together all respondents from Studies 1 and 2). A model was tested in which organizational commitment mediated the relationship between basic needs and two outcomes (job satisfaction, intentions to leave). Organizational commitment was measured by using the Klein et al. Unidimensional Target-free scale (the KUT). Results supported the nomological validity of the scale. In line with our expectations, the three needs were associated with the KUT, which in turn mediated the effects of needs on the outcomes. Practical implications of findings and directions for future research are discussed.

## Introduction

Over the last decades, Self-Determination Theory (SDT; Deci and Ryan, 2000; Ryan and Deci, 2000, 2008, 2017) has become increasingly popular in several psychological domains. According to this theory, well-being, personal growth, and individual optimal functioning are strongly associated with the fulfillment of three basic psychological needs: autonomy, competence, and relatedness. Basic needs may be conceptualized as innate and essential dispositions, not hierarchically organized, that all individuals possess; their satisfaction is required to achieve well-being and psychological health. Need for autonomy is defined as an inherited desire to experience a sense of freedom and volition in making one’s choices (Deci and Ryan, 2000; see also De Charms, 1968). Need for competence refers to the desire to develop new skills and gain mastery over the environment; this need leads people to look for optimal challenges and to enhance their personal skills (Deci and Ryan, 2000; see also White, 1959). Finally, need for relatedness represents the inherited desire to be loved and cared for, to experience closeness and connection with other people (Baumeister and Leary, 1995; Deci and Ryan, 2000). Basic needs are viewed as crucial in determining intensity, duration, and direction of behavior, regardless of situations and contexts.

In the field of organizational psychology, several scales have been developed to operationalize the three needs (see, e.g., the work motivation scale; Kasser et al., 1992). A widely used instrument has been the scale of Basic Need Satisfaction at Work, a 21-item questionnaire designed for research in the field of management (BNS-W; Deci et al., 2001; Baard et al., 2004). Over the years, however, some criticisms have been raised regarding its reliability and content validity (Gagné, 2003; Greguras and Diefendorff, 2009; Van den Broeck et al., 2010, 2016). A new 18-item measure has therefore been developed: the Work-related Basic Need Satisfaction scale (W-BNS scale; Van den Broeck et al., 2010). Research has demonstrated the three-factor structure of the scale, its validity, reliability, and independence from social desirability bias.

Over the years, research relating to SDT has evidenced positive relationships between need satisfaction and a variety of crucial behaviors in organizations, such as performance (e.g., Grant, 2008; Leroy et al., 2015), job crafting (e.g., Slemp and Vella-Brodrick, 2014; Slemp et al., 2015; Van den Broeck et al., 2016), and organizational citizen behaviors (e.g., Zhang and Chen, 2013; Van den Broeck et al., 2016). In addition, researchers have focused on exploring antecedents and outcomes of need satisfaction at work.

Several studies have shown significant relationships between the satisfaction of basic needs and the personality traits of the Five Factor Model. Specifically, positive relationships were observed between basic needs and conscientiousness and agreeableness, whereas negative relationships were observed for neuroticism (e.g., Andreassen et al., 2010; Church et al., 2013; see the meta-analysis by Van den Broeck et al., 2016). Additionally, need satisfaction is negatively related to job stressors (e.g., work-family conflict, role conflict), and positively related to job resources (e.g., Reinboth and Duda, 2006; Vansteenkiste et al., 2007; Van den Broeck et al., 2010; Bartholomew et al., 2014). Overall, need satisfaction exhibits positive relationships with organizational variables, such as, social support, job autonomy, task identity, and task significance (Van den Broeck et al., 2016).

Regarding the outcomes of need satisfaction, research has highlighted positive associations of the three needs with many indicators of well-being and with job attitudes, including affective organizational commitment (AOC). Conversely, negative relationships have been found with negative affect, strain, and burnout (Van den Broeck et al., 2010, 2016; Brien et al., 2012). Overall, findings suggest that basic need fulfillment is associated with positive outcomes for both the employees (e.g., higher well-being and job satisfaction) and the organization (higher task performance; more frequent prosocial behaviors). However, some unexpected findings have been observed. When the unique effects of each need have been estimated, need for competence turned out to be negatively related to AOC and positively related to absenteeism and turnover intentions (Van den Broeck et al., 2016).

Thus, the three needs are embedded in a rich network of relationships, which includes job resources, individual difference variables, psychological well-being, job attitudes, and job behaviors. However, novel hypotheses can be proposed, aiming to expand or deepen SDT. To test these hypotheses, we need to confirm the good psychometric properties of the W-BNS scale (Van den Broeck et al., 2010) and to show its validity in different social contexts (the scale was developed and tested in Belgium, using Dutch-speaking respondents). We focused on this measure of basic needs, because it shows several psychometric superiorities over the Deci et al. (2001) scale: the W-BNS scale, in fact, is more reliable and has higher content validity (Van den Broeck et al., 2016, p. 1200). In addition, individual needs are less intercorrelated, when the Van den Broeck et al. (2016) versus the Deci et al. (2001) scale is applied, a finding which is coherent with the supposed independence of the three needs.

Regarding other measures of motivation in the organizational field, we did not use the Multidimensional Work Motivation Scale (MWMS; Gagné et al., 2015), because this scale assesses autonomous versus controlled forms of work motivation that, in SDT, are distinct factors from basic needs (for an adaptation of MWMS to the Italian language, see Battistelli et al., 2017).

In this paper, we present validation studies of the W-BNS scale, performed on employees of Italian companies (we used quantitative validation methods; see the distinction between qualitative and quantitative validities, proposed by Sartori and Pasini, 2007). In one of these studies, the nomological validity of the scale was evaluated, testing a model in which workers’ organizational commitment mediates the relationship between need gratification and positive attitudes toward one’s job, such as job satisfaction and turnover intentions.

This work was organized into three studies. In the first, the W-BNS scale was administered to two samples of employees (*N* = 143; *N* = 457), and data were analyzed to evaluate the reliability and factor structure of the scale; exploratory and confirmatory factor analyses were applied.

In Study 2 (159 employees), we assessed the construct validity of the W-BNS scale and its independence from social desirability bias. To assess construct validity, the scale was related to attributes of the organizational context, job attitude indicators, and job burnout. As organizational variables (potential antecedents of need gratification), we used the following perceptions regarding the company: it supports cohesion among employees, promotes their professional growth, and prompts autonomy in task performance. These organizational variables should be related to the satisfaction of the three needs, although to differing degrees: autonomy support, for instance, should be primarily correlated with satisfaction of need for autonomy (see hypotheses for Study 2).^1^ Four indicators were used to measure job attitudes (potential outcomes of need gratification): job satisfaction, turnover intentions, AOC (Meyer and Allen, 1997), and commitment as conceptualized in Klein et al.’s (2012, 2014) model. AOC is a mindset characterized by emotional attachment to the organization (Meyer et al., 2002); conversely, according to Klein et al. (2012, 2014), commitment is a volitional bond that reflects dedication and feelings of responsibility. Regarding burnout, we focused on emotional exhaustion (Maslach et al., 2001), corresponding to the perception of being emotionally worn out by work (see also Schaufeli et al., 1996). The satisfaction of all three needs should be related to positive job attitudes and low feelings of burnout (Van den Broeck et al., 2016).

In Study 3, to test the nomological validity of the W-BNS scale, we incorporated this measure in a conceptual network derived from Meyer and Maltin (2010) model (the sample included all participants from Studies 1 and 2). Meyer and Maltin (2010) explained the positive relationship between organizational commitment and employees’ well-being, integrating the three-component model of commitment (TCM; Meyer and Allen, 1997) with SDT. According to their model, the affective facet of organizational commitment (AOC; the desire to belong to the organization and perform one’s duties) originates from the perception that the conditions at work satisfy the needs for autonomy, competence, and relatedness. AOC, in turn, having motivating and buffering effects, is positively related to various indicators of hedonic (e.g., life satisfaction) and eudaimonic (e.g., work engagement, feelings of hope) well-being, as well as to other positive outcomes, such as job satisfaction, better performance, and lower intentions to leave. Meyer and Maltin (2010) formulated the same hypotheses for normative organizational commitment (NOC), when this mindset reflects a moral imperative (I want to stay in this organization because it is the right thing to do) rather than an external obligation.

In the model tested in Study 3, we used commitment as conceptualized by Klein et al. (2012). This definition has the following advantages: it is more concise than previous definitions; it models commitment as a unique construct, distinguished from similar constructs (e.g., identification); it is parsimonious, ruling out the need for subordinate concepts, such as different types of bonds with the organization. Klein et al. (2014) definition includes both affective and normative contents, and its measure – the Klein et al. Unidimensional Target-free scale (KUT) – is correlated with the affective and normative dimensions of the three-component model (Klein et al., 2014).

**FIGURE 1:**
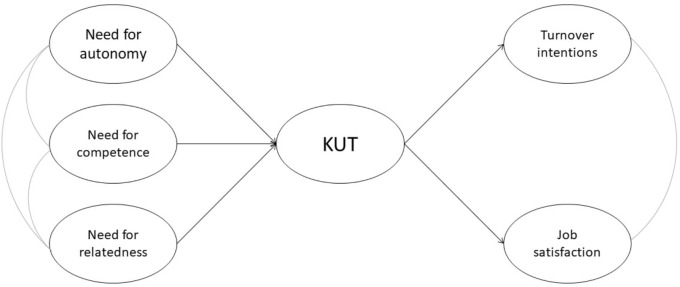
Model tested in Study 3. The curved lines indicate correlations between latent variables; KUT, Klein et al. Unidimensional Target-free scale of organizational commitment.

The KUT shows some advantages over other commitment scales: it has less overlap with measures of related constructs, like job satisfaction and turnover intentions, and its length is limited, being composed of only four items. In Study 3, we referred to Meyer and Maltin (2010) model considering Klein et al.’s (2012, 2014) commitment definition (**Figure 1**). In previous research, the association between basic needs and the KUT has been investigated by Falvo et al. (2016).

In the model of **Figure 1**, organizational commitment (measured by the KUT) mediates the association between basic needs and other job attitudes (job satisfaction, turnover intentions). Indeed, dedication to one’s organization, deriving from gratification of needs, should be related to weaker intentions to quit and greater job satisfaction. The association between the three needs and job attitudes (job satisfaction, turnover intentions) has been consistently observed in research generated by SDT (Van den Broeck et al., 2016). The association between the KUT and job attitudes (job satisfaction, turnover intentions) has been observed by Klein et al. (2014) and Cannon and Herda (2016).

The evaluation of the model of Study 3 allows a nomological validation of the W-BNS scale, because the three needs (measured by this scale) are included in a formal theoretical system: they are conceptualized as related to positive job outcomes, through the mediation of commitment (for a definition of nomological validity, see Bagozzi, 1981).

In evaluating our model (Study 3), we had a secondary goal: we aimed to test the mediation role of the KUT in the relationship between basic needs and positive job outcomes, a role that has never been previously investigated. It should be noticed that, following Van den Broeck et al. (2016) meta-analysis, we classified organizational commitment, job satisfaction, and turnover intentions as job attitudes. Indeed, AOC and the Klein et al. (2012) definition involve an evaluation of one’s organization, and job satisfaction an evaluation of one’s work (for job satisfaction, see Brief and Weiss, 2002). Regarding the intentions to leave (or stay), they are behavioral tendencies closely linked to the evaluation of one’s organization. In Study 3, we suggested a relationship between the three constructs; on the basis of previous research, we proposed that organizational commitment may be a predictor of both job satisfaction and turnover intentions (for commitment as an antecedent of job satisfaction, see, e.g., Wong et al., 1995; for commitment as an antecedent of intentions to leave, see, e.g., Tett and Meyer, 1993; Cannon and Herda, 2016).

## Study 1: Exploratory and Confirmatory Factor Analyses

In this study, the factor structure of the W-BNS scale was tested using both exploratory (EFA) and confirmatory (CFA) factor analysis. In the first step (Sample 1), EFA and PA (parallel analysis) were employed to identify poorly performing items (cross-loadings) and to define the number of factors underlying the scale. CFA was applied in the second step (Sample 2). A series of alternative factor models was evaluated as in the original work by Van den Broeck et al. (2010).

### Materials and Methods

#### Participants and Procedure

Two groups of psychology students from two large Italian Universities (North Italy) were enrolled to collect data. Students were asked to administer the research questionnaire among four of their friends or acquaintances, each selecting two women and two men, working for different organizations and belonging to different families (only a small proportion of students collected three, instead of four questionnaires). Participants were provided with an envelope containing the questionnaire and an instruction letter, where it was made clear that participation in the study was voluntary and anonymous. Participants were accurately informed about the aim of the study, the duration of the task, and the possibility of withholding their consent to participate in the research by not accepting, or accepting but not returning the questionnaire. The completed questionnaires – picked up in sealed envelopes – were returned to the researchers by the students. The project was approved by the local Ethical Committee for Psychological Research.

One group of students collected 143 completed questionnaires (Sample 1). Participants in this sample were aged between 20 and 63 (71 males; mean age = 42.28, *SD* = 12.76). The majority were office workers (54.2%, managers = 8.4%, manual workers = 9.2%, other positions = 28.2%), and different levels of working seniority were represented (up to 5 years = 36.6%, 6–10 years = 8.5%, 11–15 years = 12.0%, and 16 years or more = 42.9%). The other group of students collected 457 completed questionnaires (Sample 2). Participants were 224 males and 233 females (*N* = 457), aged between 18 and 65 (mean age = 38.10, *SD* = 13.22). Among respondents, 42.7% were office workers (managers = 2.0%, manual workers = 31.3%, other positions = 24.0%) and different levels of seniority were represented (up to 5 years = 42.4%, 6–10 years = 14.1%, 11–15 years = 12.5%, and 16 years or more = 31.0%). Participants of both samples were recruited from different professional contexts (e.g., healthcare, public service, financial sector, trading, and industry).

#### Measure

Participants completed the Italian version of the W-BNS scale (Van den Broeck et al., 2010). The scale was translated from English to Italian by two Italian investigators, and then back-translated by a native English speaker, in order to ensure linguistic equivalence. The W-BNS scale includes 18 items (six for each basic need), answered on a five-point scale (from 1 = *completely disagree* to 5 = *completely agree*). The competence subscale describes a feeling of effectiveness (e.g., “I have the feeling that I can even accomplish the most difficult tasks at work”), while the autonomy subscale describes a sense of volition and psychological freedom (e.g., “The tasks I have to do at work are in line with what I really want to do”). Finally, the relatedness subscale conveys the feeling of being loved and supported (e.g., “Some people I work with are close friends of mine”). The W-BNS scale was included in the questionnaire along with measures of other organizational constructs, such as the KUT and job satisfaction.

#### Analytic Strategies

Exploratory factor analysis and PA were conducted on Sample 1, and then a series of CFA models was tested on Sample 2. Factor analyses were run using the M*plus7* package (Muthén and Muthén, 2012), and the robust maximum likelihood as estimator (MLR; Yuan and Bentler, 2000; see also Brown, 2006; Muthén and Muthén, 2012), providing standard errors and statistical tests that are robust to non-normality.^2^EFA was applied using geomin oblique rotation; four solutions were tested with one, two, three, and four factors. PA was performed (1,000 random correlation matrices) to establish the number of factors underlying the scale.

In Sample 2, the three-factor model (autonomy, competence, and relatedness) was tested using CFA. This three-factor baseline model was matched with six alternative models: a unidimensional structure (comprising all 18 items), one two-dimensional structure (differentiating items of need satisfaction from items of need frustration), one six-factor structure (differentiating need satisfaction and need frustration for each of the three needs), and three two-dimensional structures (in which two needs were grouped into one factor, and contrasted with the third need). For the comparison between alternative models, see Van den Broeck et al. (2010).

To evaluate the models, several goodness-of-fit indices were used: χ^2^, Standardized Root Mean Square Residual (SRMR; Bentler, 1995), Root Mean Square Error of Approximation (RMSEA; Browne and Cudeck, 1993), and Comparative Fit Index (CFI; Bentler, 1990). The adequacy of a model is supported by a non-significant χ^2^, a CFI value close to 0.95 (0.90 to 0.95 for a reasonable fit), a SRMR value less than 0.08, and a RMSEA less than 0.06 (0.06 to 0.08 for a reasonable fit; see Hu and Bentler, 1999; Marsh et al., 2004). To compare non-nested models, we used the Akaike Information Criterion (AIC; Akaike, 1987) and the Bayesian Information Criterion (BIC; Schwarz, 1978), with smaller values suggesting a better fit. In evaluating the EFA models, the theoretical relevance of the solution was strongly considered.

### Results

#### Exploratory Factor Analysis

**FIGURE 2:**
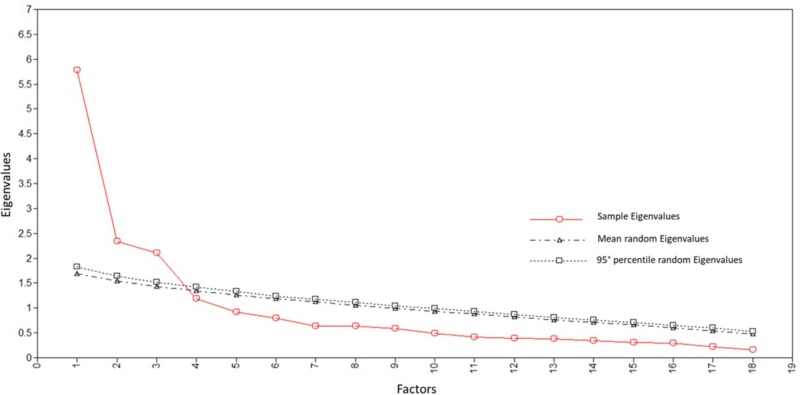
Study 1: Scree plot of real data eigenvalues, mean and 95th percentile of random data eigenvalues.

Four EFA models (Sample 1) were tested with one, two, three, and four factors. Results evidenced poor fit indices for the one- and two-factor models, whereas acceptable values were observed for the three- and four-factor models: χ^2^(102) = 192.39, *p* ≅ 0.00; RMSEA = 0.08; CFI = 0.902; SRMR = 0.05; AIC = 6,106.42; BIC = 6,364.19 (three-factor model); χ^2^(87) = 162.23, *p* ≅ 0.00; RMSEA = 0.08; CFI = 0.918; SRMR = 0.04; AIC = 6,084.82; BIC = 6,387.03 (four-factor model).^3^ In the four-factor model, however, factors were not well defined, and the fourth dimension was composed of only two items, one indicating competence and the other relatedness (“I doubt whether I am able to execute my job properly”; “I often feel alone when I am with my colleagues”). The three-factor structure, in contrast, was clearer: the three dimensions absorbed an analogous portion of variance (18.91%, 14.96%, and 14.87%); each of the 18 items loaded on the intended dimension (factor loadings ranged between 0.56 and 0.96 for competence, between 0.42 and 0.79 for autonomy, and between 0.54 and 0.77 for relatedness); and correlations between factors were moderate in size (ranging from 0.29 to 0.35). The number of factors was supported by PA (**Figure 2**). Regarding reliability, alpha coefficients for competence, autonomy, and relatedness were 0.85, 0.82, and 0.81, respectively.

#### Confirmatory Factor Analysis

Fit indices for the tested models are reported in **Table 1** (Sample 2). As shown in the table, the three-factor baseline model (competence, autonomy, and relatedness) fit the data well, and was superior to all the alternative models, which showed an inadequate fit. For the six-factor model (distinguishing need satisfaction from need frustration for each of the three needs), satisfaction and frustration factors were not distinct constructs when need for relatedness was at stake (correlation = 0.91). In fact, the 95% confidence interval, obtained considering two standard errors above and two standard errors below the observed correlation [0.79, 1.03], included 1. Correlations between the frustration and satisfaction factors were very high, but lower than 1, for the other two needs: 0.91 for autonomy, and 0.84, for competence.

**Table 1:** Study 1: Fit indices of CFA models (*N* = 457).

	χ^2^	df	*p* ≅		RMSEA	90% CI	*p* ≅		CFI	SRMR	AIC	BIC
3-factor model	332.85	132	0.00		0.06	0.05, 0.07	0.05		0.901	0.06	21,451.82	21,686.93
1-factor model	1,073.78	135	0.00		0.12	0.12, 0.13	0.00		0.539	0.11	22,263.51	22,486.24
2-factor model (AC-R)	956.60	134	0.00		0.12	0.11, 0.12	0.00		0.596	0.10	22,019.20	22,246.06
2-factor model (AR-C)	550.36	134	0.00		0.08	0.08, 0.09	0.00		0.796	0.08	21,696.16	21,923.02
2-factor model (RC-A)	726.89	134	0.00		0.10	0.09, 0.11	0.00		0.709	0.10	21,884.78	22,111.63
2-factor model (Satisfaction – Frustration)	1,061.08	134	0.00		0.12	0.12, 0.13	0.00		0.545	0.11	22,245.90	22,472.76

*RMSEA, Root Mean Square Error of Approximation; 90% CI, RMSEA 90% confidence interval; CFI, Comparative Fit Index; SRMR, Standardized Root Mean Square Residual; AIC, Akaike Information Criterion; BIC, Bayesian Information Criterion; A, need for autonomy; C, need for competence; R, need for relatedness; Satisfaction, factor measured by items indicating need satisfaction; Frustration, factor measured by items indicating need frustration.*

For the three-factor structure, all items loaded significantly on the intended factor (loadings were between 0.37 and 0.74), and correlations between factors were from medium to large (ranging from 0.37 to 0.55, *p*s < 0.001; **Table 2**), as suggested by the theoretical model. Alphas were 0.82, 0.81, and 0.74 for need for competence, need for autonomy, and need for relatedness, respectively.

**Table 2:** Study 1: Factor loadings and factor correlations for the CFA three-factor model (*N* = 457).

Item^a^	Competence	Autonomy	Relatedness
I really master my tasks at my job	0.65		
I don’t really feel competent in my job (R)	0.61		
I feel competent at my job	0.74		
I doubt whether I am able to execute my job properly (R)	0.58		
I have the feeling that I can even accomplish the most difficult tasks at work	0.69		
I am good at the things I do in my job	0.73		
The tasks I have to do at work are in line with what I really want to do		0.64	
At work, I often feel like I have to follow other people’s commands (R)		0.55	
I feel like I can be myself at my job		0.72	
If I could choose, I would do things at work differently (R)		0.54	
In my job, I feel forced to do things I do not want to do (R)		0.71	
I feel free to do my job the way I think it could best be done		0.69	
Some people I work with are close friends of mine			0.55
At work, I can talk with people about things that really matter to me			0.64
I don’t really mix with other people at my job (R)			0.37
I don’t really feel connected with other people at my job (R)			0.52
I often feel alone when I am with my colleagues (R)			0.65
At work, I feel part of a group			0.70
Competence ↔ Autonomy	0.37		
Competence ↔ Relatedness	0.36		
Autonomy ↔ Relatedness	0.55		

*All loadings and correlations are significant, p < 0.001. Competence ↔ Autonomy, correlation between need for competence and need for autonomy; Competence ↔ Relatedness, correlation between need for competence and need for relatedness; Autonomy ↔ Relatedness, correlation between need for autonomy and need for relatedness. (R), reversed item. ^a^Items are reported with the permission of authors (Van den Broeck et al., 2010).*

Thus, findings of exploratory and confirmatory factor analyses highlighted the good psychometric properties of the Italian version of the W-BNS scale. In addition, they confirmed the superiority of this scale over the Deci et al. (BNS-W; 2001) scale. In fact, in our samples, factor reliabilities were all above 0.70 (the recommended threshold by Nunnally and Bernstein, 1994); in contrast, in Van den Broeck et al. (2016) meta-analysis, alpha was below 0.70 for autonomy when the Deci et al. scale was analyzed. Furthermore, disattenuated (error-free) correlations between needs were lower in our study (W-BNS scale) compared to studies in which the BNS-W scale was used (Van den Broeck et al., 2016). Lower correlations support the assumed independence of the three needs. Thus, Study 1 demonstrates that the W-BNS scale is a reliable measure, detecting distinct psychological needs (the Italian version of the scale and all data of the three studies are available from the corresponding author, upon request).

## Study 2: Construct Validation

In Study 2, another psychometric characteristic of the W-BNS scale was investigated, specifically the independence of its items from socially desirable responding (Van den Broeck et al., 2010). Social desirability has been recognized as a problem in self-report research (e.g., Ferris et al., 2008); therefore, its impact must be accurately evaluated.

In this study, to evaluate construct validity, potential antecedents and outcomes of need satisfaction were related to the three dimensions of the W-BNS scale. Three organizational variables were considered: the organization supports cohesion among employees (social support); promotes their professional growth (professional growth); and prompts autonomy in task performance (job autonomy). Based on theory and previous research (Van den Broeck et al., 2010, 2016; Bakker and Demerouti, 2017), positive relationships were expected between these organizational variables (resources) and the satisfaction of basic needs. In particular, stronger connections were expected between social support and the satisfaction of the need for relatedness, and between job autonomy and the satisfaction of the need for autonomy. For professional growth, reliable correlations were expected with all the three needs.

Four variables were chosen to assess job attitudes: job satisfaction, turnover intentions, and two organizational commitment measures. The first assessed the affective component of commitment (AOC; Meyer and Allen, 1997), whereas the second was the KUT. In accordance with SDT, Meyer and Maltin (2010) model, and previous research (e.g., Van den Broeck et al., 2010, 2016), positive associations were expected between the satisfaction of basic needs and positive outcomes (commitment and job satisfaction), whereas negative relationships were expected between need satisfaction and turnover intentions. A measure of burnout was also used; we focused on emotional exhaustion (Maslach et al., 2001), and predicted a negative relationship between this variable and the fulfillment of the three needs.

### Materials and Methods

#### Participants and Procedure

Data were collected from two companies in the water service sector (North Italy). Questionnaires were distributed to participants in sealed envelopes in collaboration with the human resources departments. Participation was voluntary and anonymity was guaranteed. Participants were informed about the aim of the study, the duration of the task, and the possibility of not giving their consent to participate in the research, or withholding it by not returning the questionnaire. Respondents who completed the questionnaires inserted them in boxes located in common areas of the company buildings. The research project was approved by the local Ethical Committee for Psychological Research. One hundred and fifty-nine questionnaires were collected (response rate was 43.67%). Most participants were male (*N* = 159; 125 males), manual workers (47.3%), aged over 40 (61.5%), and with a working seniority between 11 and 20 years (40.1%). Office workers and specialized technicians represented 31.5% and 21.2% of respondents, respectively.

#### Measures

The Italian version of the W-BNS scale (Van den Broeck et al., 2010) was administered along with eight other measures (alpha coefficients were 0.81, 0.81, and 0.82, for need for autonomy, competence, and relatedness, respectively). The measures used were the following.

The impression management (IM) subscale of the Balanced Inventory of Desirable Responding (Paulhus, 1991) was employed to assess social desirability. This scale detects the conscious inclination to provide positively inflated self-descriptions. We applied the abbreviated Italian version elaborated by Bobbio and Manganelli (2011). This includes eight items (e.g., “I always obey laws, even if I’m unlikely to get caught”; “I have taken sick-leave from work or school even though I wasn’t really sick,” reverse coded), answered on a six-point scale (from 1 = *strongly disagree* to 6 = *strongly agree*); the alpha coefficient was 0.65.

Employees’ perceptions of organizational variables were measured using 12 items, answered on a seven-point scale (from 1 = *definitely false* to 7 = *definitely true*). Social support was assessed with five items, for instance: “In this company, people tend to get along with each other.” Job autonomy was assessed with three items (e.g., “In this company, employees have the opportunity to make decisions about their work”); four items were used to assess support for professional growth (e.g., “In this company, there is little interest in employees’ professional growth”). Alphas were 0.77, 0.70, and 0.82, for social support, job autonomy, and professional growth, respectively.

Also for job attitudes (job satisfaction, turnover intentions, and organizational commitment) a seven-point scale was used, anchored by *definitely false* (1) and *definitely true* (7). Job satisfaction was measured with six items (Dazzi et al., 1998); a sample item is “I feel satisfied with my work” (alpha = 0.82). Three items were used to assess turnover intentions, for instance: “I often think to leave this organization” (alpha = 0.80). Organizational commitment was measured with two scales: the AOC scale (Meyer et al., 1993), and the KUT (Klein et al., 2014). The AOC scale includes six items, for instance: “This organization has a great deal of personal meaning for me” (alpha = 0.91); one of the four items of the KUT was “I am dedicated to this organization” (alpha = 0.81).

To assess exhaustion, we applied five items included in the Maslach Burnout Inventory – General Survey (MBI-GS; Schaufeli et al., 1996); a sample item is “I feel exhausted by my work.” Participants were invited to indicate how often they experienced the feelings or opinions described by each item on a seven-point scale (1 = *never*, 2 = *rarely/a few times a year or less*, 3 = *occasionally/once a month or less*, 4 = *regularly/a few times a month*, 5 = *frequently/once a week*, 6 = *very frequently/a few times a week*, 7 = *daily*; alpha = 0.89).

#### Analytic Strategies

A series of CFA models was run to evaluate the effects of social desirability and the construct validity of the W-BNS scale. Models were tested using M*plus7* (Muthén and Muthén, 2012) and the maximum likelihood mean adjusted as estimator (MLM; Muthén and Muthén, 2012; see also Brown, 2006), which is robust against non-normality.^4^ To evaluate the CFA models, we used the same fit indices as in Study 1.

In line with Van den Broeck et al. (2010), the procedure suggested by Williams and Anderson (1994) was applied to explore the effects of social desirability. Two CFA models were tested, including four factors: the three basic needs and a fourth dimension, IM. Correlations of IM with the other factors were set to zero. In the first model, each item was loaded only on the respective factor; in the second model, items measuring the basic needs were loaded both on the respective factor and on the IM factor. To compare the two models, the Δχ^2^ (chi-square difference) test, developed by Satorra and Bentler (1994, 2001), was employed. Model equivalence is supported by a non-significant chi-square difference. To compare the models, we also used AIC and BIC (information criteria), and the difference between CFIs (Cheung and Rensvold, 2002): equivalence is demonstrated by a ΔCFI less or equal to 0.01.

To evaluate the construct validity of the W-BNS scale, two CFAs were run, and the relationships of need satisfaction with organizational resources, job attitudes, and exhaustion were explored. Specifically, in one CFA six factors were modeled: the three needs and the three organizational variables (social support, job autonomy, and professional growth). In the other CFA, eight factors were modeled: the three needs, job satisfaction, turnover intentions, the two commitment measures (AOC and the KUT), and exhaustion. In the two CFAs, each latent variable was measured by two parcels (for turnover intentions and job autonomy, indicators were one item and one parcel), and factors were allowed to correlate (parcels were built using the random assignment method; Little et al., 2002). To demonstrate construct validity, correlations between factors measuring basic needs and factors measuring job resources or job attitudes (and burnout) were inspected. Reliability was estimated using alpha and composite reliability (CR); the recommend cut-off value for CR is above 0.60 (Bagozzi and Yi, 1988; Bentler, 2009).

### Results

#### Method Effects, Factor Structure, and Reliability

As mentioned before, two four-factor CFA models were tested to evaluate social desirability effects. In the first model (non-confounded), the 18 items measuring basic needs were only loaded on the respective factor; in contrast, in the second model (confounded) they were also loaded on the IM factor. Both models reached a successful fit. For the non-confounded model, fit indices were: χ^2^(296) = 389.90, *p* ≅ 0.00; RMSEA = 0.05; CFI = 0.908; SRMR = 0.08; AIC = 11,322.50; BIC = 11,571.08. For the confounded model, fit indices were: χ^2^(278) = 371.49, *p* ≅ 0.00; RMSEA = 0.05; CFI = 0.909; SRMR = 0.07; AIC = 11,339.86; BIC = 11,643.69. The Δχ^2^ test supported their equivalence: Δχ^2^(18) = 18.19, *p* = 0.44 (see also ΔCFI = 0.001). In addition, in the confounded model, factor loadings of W-BNS items on the intended factors were high and significant (ranging between 0.43 and 0.79), and only two items showed a significant loading on the social desirability dimension (loadings < 0.25, *p*s < 0.05). Thus, results suggest that IM does not confound the answers on W-BNS items.^5^

Findings related to the non-confounded model supported the three-factor structure of the basic need scale. They also showed that the three factors were not overlapping constructs. In fact, for each of the three correlations – ranging from 0.09 (*p* < 0.31; competence with relatedness) to 0.58 (*p* < 0.001; autonomy with relatedness) – the 95% confidence interval never included the perfect correlation.^6^ CRs were 0.81, 0.83, and 0.82, for autonomy, competence, and relatedness, respectively.

#### Construct Validity

To evaluate the hypothesized relationships between basic needs and organizational variables, we tested a six-factor CFA model. Latent constructs were the three needs and the three job resources (**Table 3**). CFA provided the disattenuated (error-free) correlations between needs and job resources, and allowed us to test whether basic needs and organizational resources are distinct constructs.

**Table 3:** Study 2: Correlations between factors representing the three basic needs and factors representing organizational variables, job attitudes, and burnout (*N* = 159).

	CR	Need for autonomy	Need for competence	Need for relatedness
**Organizational variables (job resources)**				
				
1. Social support	0.82	0.40^∗∗∗a^	0.10^b^	0.71^∗∗∗c^
		(0.08)	(0.09)	(0.06)
2. Job autonomy	0.76	0.61^∗∗∗a^	0.25^∗∗b^	0.36^∗∗∗b^
		(0.08)	(0.09)	(0.10)
3. Professional growth	0.85	0.63^∗∗∗a^	−0.02^b^	0.41^∗∗∗a^
		(0.06)	(0.08)	(0.08)
**Job attitudes and burnout**				
1. Job satisfaction	0.89	0.86^∗∗∗a^	0.20^∗b^	0.52^∗∗∗c^
		(0.03)	(0.09)	(0.08)
2. Turnover intentions	0.83	−0.64^∗∗∗a^	−0.15^†b^	−0.42^∗∗∗a^
		(0.07)	(0.09)	(0.08)
3. Affective commitment	0.91	0.70^∗∗∗a^	0.18^∗b^	0.44^∗∗∗a^
		(0.05)	(0.09)	(0.09)
4. KUT	0.91	0.58^∗∗∗a^	0.32^∗∗∗b^	0.30^∗∗∗b^
		(0.06)	(0.09)	(0.10)
5. Exhaustion (burnout)	0.91	−0.61^∗∗∗a^	−0.04^b^	−0.41^∗∗∗a^
		(0.06)	(0.08)	(0.08)

*CR, composite reliability; KUT, Klein et al. Unidimensional Target-free scale of organizational commitment. Different letters in the same line indicate that, when the Δχ^2^ is used, the difference between the two correlations is significant, p < 0.05 (standard errors are reported in parentheses). Conclusions drawn from Δχ^2^ are supported by the application of other model comparison measures (e.g., ΔCFI), with the exception of the following cases: job autonomy-need for autonomy correlation versus job autonomy-need for relatedness correlation; job satisfaction-need for autonomy correlation versus job satisfaction-need for relatedness correlation; the KUT-need for autonomy correlation versus the KUT-need for relatedness correlation. For these cases, the difference, detected by using Δχ^2^, was not supported by other indices. Asterisks indicate that the correlation is significant. ^†^p = 0.09; ^∗^p < 0.05; ^∗∗^p < 0.01; ^∗∗∗^p ≤ 0.001.*

The six-factor model showed a good fit to the data: χ^2^(39) = 42.59, *p* ≅ 0.32; RMSEA = 0.02; CFI = 0.995; SRMR = 0.03; AIC = 8,303.43; BIC = 8,459.94. We first inspected whether the constructs corresponding to the three needs were distinct from those corresponding to job resources. Correlations between need factors and job-resource factors (**Table 3**) were generally medium or large; only two correlations, regarding competence, were non-significant. Notably, for all nine cases, the 95% confidence interval did not include the perfect correlation. This finding indicated the distinction between the two sets of constructs. Also the three organizational characteristics were distinct latent constructs: correlations were between 0.40 and 0.49, *p*s < 0.001, and 95% confidence intervals did not include 1.

Regarding the hypothesized relationships, as expected, perceived support was highly correlated with the satisfaction of need for relatedness (correlation = 0.71, *p* < 0.001). To test whether this correlation was higher than the correlation between social support and need for autonomy (0.40, *p* < 0.001), an alternative CFA model was evaluated, in which the two correlations were constrained to be equal. The difference between the two chi-squares was significant (Δ*χ*^2^ = 16.07, *df* = 1, *p* < 0.001), this finding indicating lack of equality. Lack of equality was also shown by ΔCFI, which was 0.013, and the fact that AIC and BIC were higher in the equality constrained model (AIC = 8,312.40, BIC = 8,465.95). This procedure was used to compare the three needs for each job resource. From **Table 3**, it appears that, as predicted, social support was more strongly correlated with the satisfaction of need for relatedness, and job autonomy was more strongly correlated with the satisfaction of need for autonomy than with the satisfaction of other needs. The hypothesis regarding professional growth was only partially confirmed; in fact, this job resource was only correlated with two of the three needs. Need for competence was the motive showing the least number of reliable relationships. All conclusions drawn from Δ*χ*^2^ were supported by the application of the other comparison measures, with one exception: the job autonomy-autonomy need correlation was not different from the job autonomy-relatedness need correlation, when ΔCFI and BIC were applied.

To detect the correlations between basic needs and job attitudes (and burnout) we tested an eight-factor CFA model. Latent variables were the three needs, exhaustion, and the indicators of job attitudes (job satisfaction, turnover intentions, affective commitment, and the KUT). The eight-factor model showed a good fit: χ^2^(76) = 91.74, *p* ≅ 0.10; RMSEA = 0.04; CFI = 0.990; SRMR = 0.03; AIC = 11,092.43; BIC = 11,325.67. We thus inspected whether the latent constructs expressing basic needs were distinct from those expressing job attitudes and exhaustion. Correlations between factors (**Table 3**) were generally medium or large. Only those relating to need for competence were small or medium; in addition, one correlation was non-significant (with exhaustion) and another marginally significant (with turnover intentions). For all correlations, the 95% confidence interval did not include 1, this finding demonstrating the distinction between the two sets of constructs. Also the five potential outcomes of need satisfaction were distinct constructs: the highest correlation, in absolute terms, was –0.77 (between AOC and turnover intentions, *p* < 0.001), and the lowest was –0.34 (between the KUT and exhaustion, *p* < 0.001); no 95% confidence interval included 1.

Regarding the relationships between need gratification and job attitudes (**Table 3**), as predicted, all three needs were positively correlated, albeit to different degrees, with job satisfaction and organizational commitment, whereas they were negatively correlated with turnover intentions (the correlation of need for competence with intentions to leave was marginally significant, *p* = 0.09). With the exception of need for competence, basic need gratification was associated with lower levels of emotional exhaustion. Also the correlations between the indicators of job attitudes and basic needs were compared using the chi-square difference test. For instance, the difference between the two correlations – job satisfaction with need for autonomy and job satisfaction with need for competence – was estimated comparing the baseline CFA model (eight factors) with the model in which the two correlations were constrained to equality. All conclusions drawn from Δχ^2^ were supported by at least two of the other comparison measures, with only two exceptions: the job satisfaction-need for autonomy correlation was not different from the job satisfaction-need for relatedness correlation, when ΔCFI and BIC were applied; the same result was obtained when the KUT-need for autonomy correlation was compared with the KUT-need for relatedness correlation. All measures used showed satisfactory composite reliability (**Table 3**).

The results of this study support the validity of the Italian version of the W-BNS scale. Analyses suggest that social desirability does not confound the answers. Furthermore, confirming SDT, basic need satisfaction was associated with relevant job resources, employees’ well-being (low burnout), and positive job attitudes. Weaker relationships were observed for need for competence, supporting the findings of Van den Broeck et al. (2016; Table 5). As noted by these authors, employees who feel competent think they can be appreciated by different employers; accordingly, they feel less committed to the organization, and are more inclined to look for work elsewhere. Thus, the W-BNS scale turns out to be a valid tool in two languages and cultural contexts.

Our results suggest future research and practical interventions. We found that basic need gratification is related to job resources: social support, job autonomy, and support for employee professional growth. Future longitudinal studies, performed in the two water-service or similar companies, could highlight the causal links between resources and needs, allowing interventions aimed to improve employee motivation with its positive outcomes.

## Study 3: Testing the Nomological Validity of the W-Bns Scale

In Study 3, we evaluated a model (**Figure 1**), based on Meyer and Maltin (2010) model, in which organizational commitment, measured by the KUT (Klein et al., 2014), mediates the relation between basic needs and two other job-attitude indicators: turnover intentions and job satisfaction.

### Materials and Methods

#### Participants and Procedure

A sample of 759 (420 males) participants was formed by grouping all respondents from Study 1 and Study 2. Participants completed the W-BNS scale and the KUT, along with the measures of turnover intentions and job satisfaction. Most participants were manual workers (30.2%) or office workers (42.7%; managers = 2.8%; other positions = 24.3%). Regarding age, the most frequent categories were over 40 years (53.2%) and up to 30 years (29.5%). For length of service, the most frequent categories were up to 10 years (49.8%) and over 20 years (25.9%). Participation in the survey was voluntary and anonymous (see the “Participants and Procedure” sections, in Study 1 and Study 2; the two sections provide information also with respect to response rate).

#### Measures

Regarding the Italian version of the W-BNS scale, in the whole sample (*N* = 759), alphas were 0.81 for need for autonomy, 0.82 for need for competence, and 0.77 for need for relatedness. Organizational commitment was assessed using the KUT (Klein et al., 2014; alpha = 0.81). With respect to the outcomes, as in Study 2, job satisfaction was assessed using the six-item scale by Dazzi et al. (1998; alpha = 0.86), and turnover intentions using the three-item scale (alpha = 0.82). The answer format was on a seven-point scale (from 1 = *definitely false* to 7 = *definitely true*); it was on a five-point scale (from 1 = *completely disagree* to 5 = *completely agree)*, for the scale of basic needs.

#### Analytic Strategies

**FIGURE 3:**
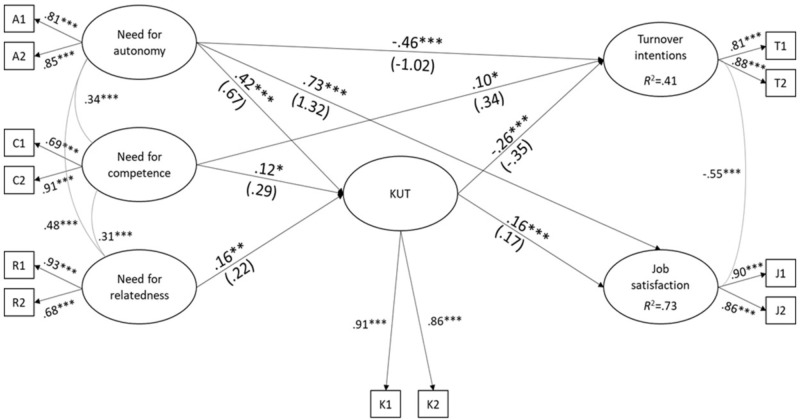
Study 3: Test of the model of **Figure 1**. The curved lines indicate correlations between latent variables; KUT, Klein et al. Unidimensional Target-free scale of organizational commitment. Unstandardized path coefficients in parentheses (*N* = 759). ^∗^*p* < 0.05; ^∗∗^*p* < 0.01; ^∗∗∗^*p* < 0.001.

A structural equation model with six latent variables was evaluated (**Figure 3**), and to test mediation the direct paths from basic needs to the outcomes were estimated. All constructs were measured using two parcels as indicators (parcels were created applying the random assignment method; Little et al., 2002), and the model was tested considering MLM (maximum likelihood mean adjusted) as estimator (Muthén and Muthén, 2012; see also Brown, 2006). The significance of indirect effects was assessed employing bootstrapping procedures (5,000 resamples) and 95% confidence intervals. To evaluate the adequacy of the overall structural model, we used *χ*^2^, RMSEA, CFI, and SRMR (see Study 1).^7^

### Results

#### Test of the Model

The tested model reached an adequate fit: χ^2^(39) = 65.23, *p* ≅ 0.005; RMSEA = 0.03; CFI = 0.993; SRMR = 0.02.^8^ As shown in **Figure 3**, the satisfaction of all three needs was positively related to the KUT, which in turn was positively related to job satisfaction and negatively related to turnover intentions. The association of need for autonomy with both outcomes was both direct and mediated by the KUT. The 95% bootstrap confidence interval was [0.06, 0.19], point estimate = 0.12 (unstandardized coefficients), for the indirect effect on job satisfaction. For the indirect effect on turnover intentions, it was [–0.36, –0.15], point estimate = –0.24. The reliability of indirect effects was demonstrated by the fact that the two CIs excluded zero.

With regard to need for relatedness, only the indirect effects were significant (**Figure 3**). The 95% bootstrap confidence intervals were [0.01, 0.08], point estimate = 0.04, for job satisfaction, and [–0.15, –0.03], point estimate = –0.08, for turnover intentions. The two effects were also significant for need for competence: the confidence intervals were [0.01, 0.12], point estimate = 0.05, for job satisfaction, and [−0.22, −0.02], point estimate = −0.10, for turnover intentions. Also a direct (and positive) path linked need for competence to turnover intentions (**Figure 3**). Findings thus support the hypothesized mediation role of the KUT.

Study 3 allowed us to demonstrate the nomological validity of the W-BNS scale. In fact, using this scale, we were able to support basic hypotheses included in Meyer and Maltin (2010) model. It should be observed how the findings of Study 3 replicated those of Study 2: in both cases, basic needs were positively associated with the KUT and job satisfaction, and negatively associated with turnover intentions (in Study 3, however, the relationship of need for competence with intentions to leave was both positive and negative). Interestingly, the associations observed in Study 3 were unique, that is, obtained for each need controlling for the effects of the other needs.

#### Alternative Models

Although the hypothesized model was supported by data, we tested alternative models. In one model (Model A), turnover intentions and job satisfaction were the mediators, and the KUT was modeled as the outcome. In the other alternative model (Model B), the KUT was the independent variable, and basic needs were the mediators; turnover intentions and job satisfaction were the outcomes. In both cases, to test mediation, also the direct paths from the exogenous variables to the outcomes were estimated. The baseline and the alternative models are therefore equivalent with same goodness-of-fit indices (for the concept of equivalent models, see Tomarken and Waller, 2003). Hence, the three models have to be compared on the basis of theory and previous research.

Findings regarding Model A confirmed the positive relationship between basic needs and the KUT: need for competence and need for relatedness were directly associated with the KUT, whereas need for autonomy was associated with the KUT through the mediation of lower intentions to leave. In this model, however, contradicting SDT and previous findings (Van den Broeck et al., 2010, 2016), need for competence did not show significant relationships with job satisfaction and intentions to quit. Also findings regarding Model B confirmed the positive association between organizational commitment and the three needs; however, in this model, contradicting SDT and previous evidence (Van den Broeck et al., 2010, 2016), need for relatedness did not show significant relationships with job satisfaction and turnover intentions (findings are available from the first author, upon request).

Hence, our baseline model was the most consistent with theory and existing evidence. It should be noted that, in the baseline model, basic needs were reliable antecedents of the KUT, whereas in Model B, the KUT was a reliable antecedent of basic needs. Probably, the relationship between needs and organizational commitment is reciprocal. Longitudinal studies are needed to verify this hypothesis of bidirectionality.

## Discussion

In this work, we validated the W-BNS scale in the Italian social context. Three studies were performed. In Study 1, two samples of employees, working for various organizations, were examined. Both exploratory and confirmatory factor analysis supported the three-dimensional structure of the scale. Findings also showed that the three subscales measured distinct constructs.

Results of Study 2, performed in two companies in the water service sector, showed that social desirability does not confound the answers on the W-BNS items. In addition, supporting SDT and, hence, the construct validity of the scale, we noticed that the three needs were associated with relevant job resources, low burnout, and positive job attitudes. However, weaker relationships were observed for need for competence than for the other needs.

The sample for Study 3 included all participants examined in Study 1 and Study 2. Results supported the nomological validity of the scale. We found that the three needs (measured by the W-BNS scale) were included in a conceptual network, consistent with Meyer and Maltin (2010) model. They were associated with organizational commitment, and with positive outcomes (job-satisfaction, lower intentions to leave), through the mediation of commitment. Consistent with SDT, each need was uniquely associated with commitment and the outcomes.

In testing our mediation model, we also observed direct effects of need for autonomy on both outcomes. It is likely that organizational commitment, using Klein et al. (2014) definition, does not absorb all the positive influences of autonomy need satisfaction, which has many energizing effects such as work engagement and creative performance (Van den Broeck et al., 2016; Table 7).

For need for competence, its direct, positive association with turnover intentions may indicate that employees who feel competent may be inclined to look for new job opportunities. However, turnover intentions, linked to competence perceptions, are partially curbed by feelings of dedication and responsibility (the KUT) toward one’s organization (for the positive association of the need for competence with turnover intentions, see also Van den Broeck et al., 2016; Table 7).

Regarding the relation of need for competence with organizational commitment, obtained controlling for the effects of other needs, it was positive in Study 3 (**Figure 3**), but negative in Van den Broeck et al. (2016) meta-analysis. We believe that this different finding depends on the commitment scale (and concept) used. Probably, the satisfaction deriving from being able to show one’s capacities leads more to feelings of responsibility (KUT measure: our study) than to a general emotional attachment (AOC measure: Van den Broeck et al., 2016).

Overall, findings demonstrate the good psychometric properties of the Italian version of the scale. Future research should clarify why need for autonomy is more strongly related than the other needs to organizational commitment and job satisfaction (Study 2), and why need for competence exhibits a positive, direct link with withdrawal intentions (Study 3; similar findings are reported by Van den Broeck et al., 2016). The stronger impact of need for autonomy could be due to cultural factors. As noted by Van den Broeck et al. (2016), employees from collectivistic cultures may benefit more from the satisfaction of need for relatedness, whereas employees from individualistic cultures may benefit more from the satisfaction of need for autonomy. More research examining cultural differences is needed.

In testing our mediation model (Study 3), we made reference to Klein et al. (2012) definition of organizational commitment and to the corresponding measure. As noted in the introduction, the first is more parsimonious than Meyer and Allen (1997) definition, while including both affective and normative components. Regarding the KUT, it has the advantage of showing less overlap with correlated constructs (e.g., job satisfaction) than other commitment scales. Findings, showing the mediation role played by the KUT, support the use of this measure (and related concept) in testing hypotheses drawn from SDT and Meyer and Maltin (2010) model.

Study 3’s findings have practical implications: in any organizational setting, increasing need gratification by enhancing job resources (e.g., perceived firm support) may lead to stronger commitment and, thus, to greater job satisfaction and weaker intentions to leave. Findings have, however, the usual limitation of cross-sectional research, which does not allow a temporal ordering of causal variables. In future studies, our model should be tested using longitudinal designs. Future studies should also consider other outcomes, for instance, indicators of hedonic and eudaimonic well-being (Meyer and Maltin, 2010), and not only self-report data, but also more objective measures, such as, indicators of short-term or long-term variations in health. Another direction for future research with the W-BNS scale could consider the relationship between needs and employees’ age. Satisfaction of need for relatedness, for instance, could be more important for older employees, whereas satisfaction of need for competence could be more important – and affect job attitudes more – for younger employees.

## Author Contributions

DoC contributed in designing the studies. RF adapted the questionnaires and directed the research. DaC and GDB performed the statistical analyses. DaC wrote the first draft of the manuscript. All authors contributed to manuscript revision, read and approved the submitted version.

## Conflict of Interest Statement

The authors declare that the research was conducted in the absence of any commercial or financial relationships that could be construed as a potential conflict of interest.The reviewer EM and handling Editor declared their shared affiliation.

## Footnotes

1 In the context of Job Demands-Resources (JD-R) theory (Bakker and Demerouti, 2007, 2017), the three organizational variables correspond to job resources, that is, they are psychological, social, or organizational aspects of the job, which are functional in achieving one’s work goals. Job resources reduce job demands’ costs and promote personal growth.

2 Normality was investigated for both the EFA and CFA datasets through the inspection of univariate distribution of each variable (Kolmogorov–Smirnov test; K–S) and using multivariate normality tests (Mardia’s tests of skewness and kurtosis, and the test HZ of Henze and Zirkler, 1990). Both datasets showed departure from univariate (K–S ranging from 0.16 to 0.29, *p*s < 0.001) and multivariate normality. Regarding multivariate normality, for EFA dataset, HZ was 1.02, *p* < 0.001, and Mardia’s values were 1,769.73 (multivariate skewness) and 8.47 (multivariate kurtosis), *p*s < 0.001; for CFA dataset, HZ was 1.07, *p* < 0.001, and Mardia’s values were 2,531.64 (multivariate skewness) and 22.53 (multivariate kurtosis), *p*s < 0.001. The adequacy of data to factor analysis was shown by Kaiser–Meyer–Olkin coefficient and Bartlett’s test: Kaiser–Meyer–Olkin (KMO) coefficients were 0.83 for EFA and 0.86 for CFA; both Bartlett’s tests were significant, *p*s < 0.001.

3 Fit indices for the one-factor model were: χ^2^(135) = 615.98, *p* ≅ 0.00; RMSEA = 0.16; CFI = 0.477; SRMR = 0.13; AIC = 6,476.22; BIC = 6,636.21. Fit indices for the two-factor model were: χ^2^(118) = 463.73, *p* ≅ 0.00; RMSEA = 0.14; CFI = 0.624; SRMR = 0.09; AIC = 6,260.34; BIC = 6,470.70.

4 Normality distribution for each variable and multivariate normality were checked, like in Study 1. Overall, results showed departure from normality: K–S ranged from 0.07 to 0.45, *p*s < 0.02; HZ ranged from 1.00 to 1.06, *p*s < 0.001; Mardia’s estimations ranged from 564.02 to 1,197.98 (*p*s < 0.001), for multivariate skewness, and from 6.30 to 9.52 (*p*s < 0.001), for multivariate kurtosis. KMO coefficients (from 0.74 to 0.86) and Bartlett’s tests (*p*s < 0.001) supported the adequacy of data to factor analyses.

5 Also the AIC and BIC measures showed the independence of the W-BNS scale from social desirability factors; the two indices, in fact, were lower for the baseline (non-confounded) model than the confounded model.

6 The confidence intervals were: [–0.09, 0.27], for competence with relatedness; [0.03, 0.39], for competence with autonomy; and [0.45, 0.71], for autonomy with relatedness.

7 Normality distribution for each variable and multivariate normality were checked. Results revealed departure from normality: K–S ranged from 0.08 to 0.20, *p*s < 0.001; HZ was 1.26, *p* < 0.001; Mardia’s estimation was 1,131.51, *p* < 0.001, for multivariate skewness, and 23.83, *p* < 0.001, for multivariate kurtosis.

8 Before testing the structural equation model of **Figure 3**, we evaluated the conceptual distinction between the six latent variables. A CFA model was tested. Findings showed that the six factors were correlated (*p*s ≤ 0.001), but distinct constructs. For each correlation, in fact, the 95% confidence interval excluded the perfect correlation. CRs ranged from 0.78 to 0.88.
